# Refining Vegetable Oils: Chemical and Physical Refining

**DOI:** 10.1155/2022/6627013

**Published:** 2022-01-11

**Authors:** Said Gharby

**Affiliations:** Laboratory Biotechnology, Materials and Environment (LBME), Polydisciplinary Faculty of Taroudant, Ibn Zohr University, Agadir, Morocco

## Abstract

This review presents recent technologies involved in vegetable oil refining as well as quality attributes of crude oils obtained by mechanical and solvent extraction. Usually, apart from virgin oils, crude oils cannot be consumed directly or incorporated into various food applications without technological treatments (refining). Indeed, crude oils like soybean, rapeseed, palm, corn, and sunflower oils must be purified or refined before consumption. The objective of such treatments (chemical and physical refining) is to get a better quality, a more acceptable aspect (limpidity), a lighter odor and color, longer stability, and good safety through the elimination of pollutants while minimizing oil loss during processing. However, the problem is that refining removes some essential nutrients and often generates other undesirable compounds such as 3-MCPD-esters and trans-fatty acids. These compounds directly influence the safety level of refined oil. Advantages and drawbacks of both chemical and physical refining were discussed in the light of recent literature. Physical refining has several advantages over chemical one.

## 1. Introduction

**Figure 1:**
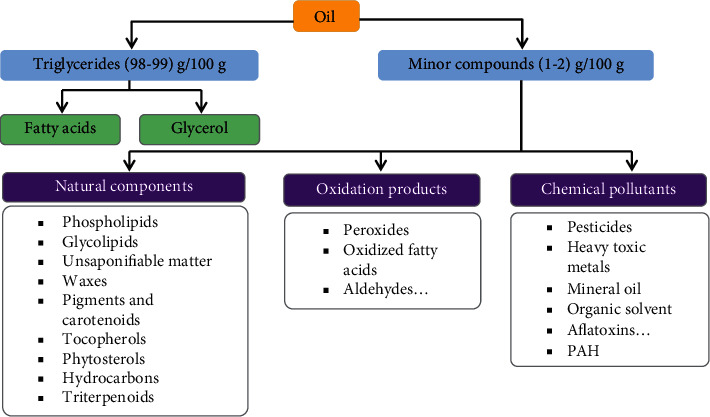
General overview of chemical composition and contaminants of vegetable oils.

Vegetable oils and fats are important constituents of foods and are essential components of our daily diet [1]. Vegetable oils are obtained by mechanical expelling or solvent extraction of oleaginous seeds (soybeans, rapeseed, sunflower, etc.) or oleaginous fruit like palm and olive [2]. Vegetable oils generally contain triglycerides (about 98 g/100 g) [3], triesters resulted from a reaction between glycerol and fatty acids, and other substances in a minority proportion (Figure 1) [4]. Some of them such as diglycerides, vitamins, phytosterols, tocopherols, and polyphenols have important health benefits in humans [5, 6], and therefore they should not be removed during processing. Other compounds known for their negative effect on the quality and stability of oils, include free fatty acids, unsaponifiable matters, waxes, pigments, solid impurities (mainly fibers), oxidation products (peroxides, aldehydes, ketones, alcohols, and oxidized fatty acids) [7, 8] (Figure 1).

These compounds are not toxic, but their presence in oils is undesirable as they affect the stability and the sensorial acceptability of consumers. Indeed, these molecules can give bad taste and smell and could affect the functional properties of the oil [9–11]. Vegetable oils can also contain some contaminants: pesticides, trace metals, mineral oil aromatic hydrocarbons (MOAH), aflatoxins, dioxins, polycyclic aromatic hydrocarbons (PAH) [12, 13], and organic solvent traces, [14, 15]. The origin of these pollutants could be attributed to the environment under which oleaginous crops are grown, seeds are transported and stored, and crude oils are processed and stored [15]. Nowadays, there is a global pressure by both consumers and food industries regarding refined oil quality to meet the established specifications for food safety [16, 17]. The oil must be odourless and rather neutral in taste, limpid, and colourless, and it must be free of contaminants [10].

Treatment that eliminates undesirable and toxic components in crude oils is known as “refining” [9]. Refining is practically mandatory for crude oils that cannot be consumed as virgin oils to provide a product with an attractive appearance, a neutral taste, and more resistance to oxidation. Likewise, it allows obtaining oils that are more suitable for various industrial uses and getting rid of undesirable substances such as pesticide residues, metal traces, polycyclic aromatic hydrocarbons, dioxin, and alteration products as well as minimizing oil loss during processing (Table 1) [11].

Although refining extends oil shelf life, it has several disadvantages. One of the main disadvantages is the loss of substances responsible for healthy, pharmaceutical properties and technological interest in the oils, such as tocopherols, phospholipids, squalene, polyphenols, and phytosterols [5, 18]. Another notable disadvantage of refining is the formation of undesirable compounds such as glycidyl ester, 3-MCPD-esters [19], harmful trans-fatty acids [5, 20], and polymeric triacylglycerols [21]. These can directly influence the safety level of refined oils.

Several studies were devoted to determining the effects of refining on the minor bioactive components such as sterols and tocopherols. Indeed, Verhé et al. [22], who found a sterols loss of 10–32% (physical refining) and 13–31% (chemical refining). A similar trend was recorded by the same authors regarding tocopherols for physical (7.7–76.5 g/100 g) and chemical refining (26.8–79.4%). So, tocopherols decrease in vegetable oils substantially and directly influence decrease in the shelf life of oils and the nutritional quality [20, 22].

In this context, such refining process for crude oils should be undertaken with the aim of removing undesirable compounds and avoiding the least possible damage to desirable components [14, 23]. It is also important to minimize oil and generate less levels of harmful compounds such as 3-MCPD-esters [19, 23] and unhealthy trans-fatty acids [5]. Some review articles were devoted to describing refining process and technology involved as well as its effects on refined vegetable oils with an emphasis on chemical composition [23–26]. The present review aimed at comparing the two main industrial technologies used for vegetable oils' refining, namely, chemical refining and physical refining. Chemical refining removes free fatty acids by soda neutralization. Physical refining eliminates undesirable compounds (deacidification) by distillation under a high vacuum with steam injection [10].

### 1.1. Chemical Refining of Oil

Chemical refining is the traditional method used since ancient times. It can be used for all fats and oils even when they have been slightly degraded. Each step of the refining process has specific functions for removing some undesirable compounds. Chemical refining follows six processes:

1. Degumming with the goal of the elimination of phospholipids and mucilaginous gums [27]
2. Neutralization, which allows the elimination of free fatty acids (FFA), phospholipids, metals, and chlorophylls [23, 28]
3. Washing and drying in order to eliminate residuals of soaps and water
4. Bleaching aims at eliminating pigments, peroxides, and residuals of both fatty acids and salts [23, 27]
5. Dewaxing has as main objective removing the waxes in the case of oils rich in waxes [29]
6. The final stage of chemical refining is deodorizing, which allows the elimination of volatiles, carotenoids, and free fatty acids [30–32].

However, the chemical refining has several drawbacks as each process step participates in removing also certain bioactive molecules. These consist mainly of tocopherols and polyphenols, which can act as antioxidants [33]. Likewise, chemical refining requires higher cost and might result in the release of polluting effluents.

#### 1.1.1. Degumming

Degumming is a crucial step in the refining process of vegetable oils [9]. It allows the elimination of “gums” or “mucilage,” composed mainly of phospholipids from the crude oil as well as compounds such as carbohydrates, proteins, and trace of metals [9, 34].

Phospholipids or phosphatides are naturally present in oils. These compounds are important biochemical intermediates in the growth and functioning of plant cells [35]. Phosphatidylcholine (PC), phosphatidylethanolamine (PE), phosphatidylserine (PS), and phosphatidylinositol (PI) are the major types of phospholipids [33]. In general, vegetable oils contain two types of phospholipids: hydratable and nonhydratable [35–37].

These compounds can trap metallic ions (copper + iron) and prevent their catalytic activity related to free radical production in crude oils [38]. Moreover, the presence of these compounds in crude oils poses many problems for storage and processing. Phospholipids are often linked to heavy metals, which are catalysts in oxidation reactions and, sometimes, can act as prooxidants in vegetable oils [25]. The incomplete removal of phosphorus-rich components during alkaline neutralization creates a series of subsequent refining difficulties resulting in the formation of a dark color settling in during storage [39]. Therefore, their elimination from crude oils is mandatory. Indeed, the degumming stage consists of eliminating all compounds susceptible to becoming insoluble through hydration (phospholipids, glycolipids, proteins, etc.) [40].

There are four types of degumming processes, namely, water degumming, acid degumming, dry degumming, and enzymatic degumming. Chemical refining generally starts with the degumming step [34]. The conditioning prior to degumming consists of mixing the oil with a small quantity of acid such as phosphoric or citric acid to dissociate nonhydratable phospholipids [34]. For some oils, the first degumming with water can be done beforehand to remove hydratable phospholipids [26, 38]. The gums recovered represent the raw lecithin [10].

The dry degumming process is recommended for oils with a low phospholipid content. This technique uses a concentrated acid (phosphoric or citric) combined with bleaching earth (1 to 3 g/100 g). The acid (0.05 to 1.2 g/100 g) is dispersed in oil at 353°K (80°C). This acid dissociates the nonhydratable phosphatides into phosphatidic acid, and it is eliminated by centrifugation. The remaining amount is further adsorbed through bleaching earth. Dry degumming was developed for palm-, palm kernel-, and coconut-type oils containing small amounts of phospholipids. The degumming process combines the acid degumming step with the bleaching process, thus eliminating the water addition and centrifugation of the gums. This technique is carried out at 393 to 413°K (120–140°C) under a reduced pressure. Its main benefit is the lack of generation of aqueous effluents, except water is used in the vacuum system. The latest process in degumming is enzymatic degumming, which offers many benefits.

Enzymatic refining is a kind of biotechnological process in which a phospholipase, especially the phospholipase C, converts nonhydratable phospholipids into lysophospholipids [9, 41]. These components are insoluble in oil and need to be removed by centrifugation [42]. Enzymatic degumming is a relatively new technology that emerged in the past 20 years. The process was first reported in the 1990s by Roehm and Lurgi referring to the “EnzyMax Process” project [31, 43]. The enzyme was used to hydrolyze nonhydratable phospholipids into their hydratable form [31, 44, 45]. Since then, recent works have focused on the use and development of new enzymes to optimize the reduction of phosphorus levels [46]. During the process, crude oil is pretreated with a combination of citric acid and caustic soda. It is then mixed with water and an enzyme (Lecitase Ultra) using a high shear mixer. This creates a very stable emulsion that can be broken by centrifugation then separated into phospholipids and mucilaginous materials from the oil [45, 47]. Enzymatic degumming is a unique process, very different from acid degumming. Indeed, both hydratable and nonhydratable phospholipids present in the oil are hydrolyzed to the corresponding lysophospholipids [41].

#### 1.1.2. Neutralization

Free fatty acids content is expressed in g/100 g of oleic acid except for some oils such as palm oil where it is reckoned in g/100 g of palmitic acid, and coconut and palm kernel oils, where it is in g/100 g of lauric acid. Acidity depends on the nature of the oil, which, in turn, depends upon its geographical origin, harvesting, seed crushing conditions, and storage duration [48]. It ranges from a value below 0.7 to 10 g/100 g for some especially degraded oils.

Vegetable oils containing a high percentage of free fatty acids (by hydrolysis and/or oxidation) must be refined to be edible [49]. The presence of these compounds in crude oils poses many problems for the storage and result in an undesirable color and odor in the final product [6, 25]. Free fatty acids influence the chemical quality and the organoleptic instability of oil [5]. Many methods for fatty acids elimination have been developed to improve the value of degraded vegetable oils. Some include chemical refining with caustic soda neutralization and physical refining based on steam distillation [49, 50].

In chemical refining, the oil is treated with an alkali solution (caustic soda) that reacts with the free fatty acids (FFA) present as per the following equation and converts them into soap stock [40, 51]:

(1) $$\begin{array}{c} \text{R}-\text{COOH}\left(\text{acid}\right)+\text{NaOH}\left(\text{base}\right)⟶\text{R}-\text{COONa}\left(\text{soap}\right)+{\text{H}}_{2}\text{O}\left(\text{water}\right) \end{array}$$

In addition, when the neutralization is not properly done, the caustic soda may not only neutralize the fatty acids, which is the desired aim, but it also attacks the neutral oil in the form of a so-called “parasitic” saponification that also reduces the yield, as per equation (2), especially when crude oil acidity is low. Therefore, the concentration and dosing rate of the caustic soda need to be calculated based on the FFA of the degummed oil for optimal neutralization.

(2) $$\begin{array}{c} \text{Triglyceride}+\text{Caustic Soda}⟶\text{Soap}+\text{Glycerol}. \end{array}$$

In terms of economic perspective, caustic soda neutralization cannot be applied to oils having a percentage of free fatty acids greater than 15 g/100 g because the loss of neutral oil in soaps becomes very significant [49].

Moreover, other compounds can be removed in this step. These are an excess of phosphoric acid, residual proteins, residual gums, carbohydrates, oxidation products from FFA, traces of metals, and pigments [52], which facilitate bleaching and deodorization. Moreover, a part of some bioactive molecules in oil, which can act as antioxidants like tocopherols and polyphenols, are also removed.

Formed soap is generally insoluble in oil. Hence, it can be easily separated mechanically from oil based on the difference in specific gravity between the soap and neutral oil. The separated oil is then washed with water to remove the soap, alkali solution, and other impurities [52] to make it ready for the decolorizing or deodorizing process.

Soapstocks are quite alkaline (pH = 10–11) [53]. They contain sodium soap and caustic soda, but also water, salt, sodium phosphates, gums, carried-over neutral oil, coloring agents, oxidation by-products, and various contaminants. This residual is referred to as residual oleins [54], which are treated and valorized in a special unit.

Soapstocks have multiple end uses. These include applications in lipochemistry [34, 43], as an alternative raw material for biodiesel production [55] and as an ingredient in animal feed [56].

#### 1.1.3. Washing and Drying

This operation eliminates alkaline substances present in the oil from coming out of the neutralization turbine (caustic soda and excess soap) as well as last metallic and phospholipids traces and other impurities. The crude oil needs to be well prepared. Otherwise, sizeable emulsions could take place and part of the soap may not be eliminated. Washing water should be as hot as possible—358 to 363°K (85–90°C)—and should represent 5–15% of treated oil depending on whether the operation is done in one or two stages. It is preferable to use softened water and washing water should be sampled regularly for a visual check of the quantity of fat carried away (after natural decantation or, even better, after centrifugation).

The oil, free of gums, traces of soapstock, and other impurities, is pumped through a plate heat exchanger where it is heated by steam. It is then sent to the centrifugal mixer to be combined with water and further centrifuged in a centrifuge for water washing. After this treatment, water-washed oil is dried with a vacuum dryer until the moisture level of the oil falls below 0.1%. This stage will be followed by bleaching.

The moisture present in the water-washed oil can rapidly clog the filters, especially in the presence of soap.

#### 1.1.4. Bleaching

The bleaching is a critical step in the refining process of oils [57, 58], preceded generally by degumming, neutralization, and drying processes. Bleaching is a complex physical and chemical process employed in the refining of vegetable oils. The objective of bleaching (or decolorizing) is to reduce the levels of colored pigments (carotenoids and chlorophylls). It also further removes residue traces of phosphatide, soap, phospholipid contaminants, lipid peroxidation products, and other impurities [24, 59]. Finally, it indirectly impacts the deodorized oil color. To perform bleaching, adsorption bleaching clays, activated carbon, special silica, or a combination of these are used [57].

The bleaching earth is the most popular adsorbent for decolorization of oil and the most widely used adsorbent material by the oil industry [57, 58, 60]. Bleaching clay is favored over other adsorbents such as silica-based and activated carbon products due to its low cost and relatively high adsorption capacity [58, 60]. Indeed, bentonite is the most favored bleaching clay used in the oil industry [58].

In general, activated earth has no bleaching properties in their natural state. Their chemical composition does not indicate that they can be activated. Indeed, activation is the transformation of silicates into colloidal silica, which possess an important adsorbing power. Activation is a chemical reaction of strong inorganic acid (sulfuric or hydrochloric acids) at temperatures lying between 353°K (80°C) and 403°K (130°C). Chemical treatment significantly changes their textural characteristics [61]. Strong acids act by substituting protons for cations while increasing notably the adsorbing surface. Earth's quality depends on the amount and the nature of acid used, the contact time, and the temperature [59, 62]. The degree of bleaching is dependent upon the level of cation substitution by the hydrogen ions of the acid in the clay structure, according to the following equation [62]:

(3) $$\begin{array}{c} \text{Cation}-\text{Clay}+2{\text{H}}^{+}⟶\text{H}-\text{Clay}+\text{Cation}. \end{array}$$

For cost control reasons, sulfuric acid is preferred over hydrochloric acid. New pieces of evidence have shown that other acids (mainly phosphoric, acetic, and oxalic acid) can be used to activate bentonite clay for the removal of organic dyes (Mordant Red 73) [63]. Moreover, acid-activated bleaching earth is sometimes called bentonite. At the final stage, the activated earth is washed to eliminate, as much as possible, the acid and metallic salts resulting from the acid action. They are then dried and crushed.

Another important adsorbent (powder or granules) in the bleaching process is activated carbon; it is the perfect adsorbent to remove unwanted color or other organic contaminants such as polycyclic aromatic hydrocarbons, benzo(a)pyrene, benzo(a)anthracene, benzo(b) fluoranthene, and chrysene [64]. Polycyclic aromatic hydrocarbons (PAHs) are a class of universal chemical contaminations in vegetable oils [65]. On the other hand, activated carbon also removes residues of some polyaromatic hydrocarbons of mineral oils [57] as well as other products.

Long since used in the sugar industry, research on their adsorbing properties dates back to the First World War when they were used in gas masks. Thus, activated carbon is produced from a variety of carbon-containing substances. Activated carbon contains 95 to 98 g/100 g carbon and is characterized by its porosity. It is admitted that the specific surface of 1 g of activated carbon reaches 600 to 1000 m^2^. Activation develops the capillary structure and unclogs the pores obstructed by tars. Preparation of activated carbon comprises two main steps. The first one is carbonization, which is a thermal decomposition (temperature around 1073°K (800°C)) of the basic materials in an inert atmosphere, while the second one of the activation of the carbonized products. This activation can be performed by physical [66, 67] and/or chemical treatment [68] with the aim of expanding the diameters of the small pores and creating new pores.

To obtain a high adsorption capacity in the bleaching of some oils, a mixture of activated carbon and bleaching earth is used in refining industries. In general, the amount of activated carbon must be in the range of 5–10 g/100 g to the amount of bleaching earth. This technique is very well documented in the literature [8, 69].

The usual method of bleaching occurs through the adsorption of pigments over an adsorbent material. In general, when an absorbent comes into contact with oil, the absorbent attracts to its surface colored pigments and other compounds that need to be eliminated. The attraction condenses the molecules and they form a casing inside of which the concentration of an adsorbed substance in oil differs from the initial concentration. Langmuir's (4) and Freundlich's (5) equations theoretically give the adsorption capacity [58]:

(4) $$\begin{array}{c} \text{Langmuir }\frac{\text{Xe}}{\left(x/m\right)}=\frac{1}{a}+\frac{b}{a}\cdot \text{Xe}, \end{array}$$

(5) $$\begin{array}{c} \text{Freundlich Log}\left(\frac{x}{m}\right)=N\cdot \;\log\left(\text{Xe}\right)+\text{Log}\left(K\right), \end{array}$$

where *m* is the amount of adsorbent, *x* is the amount of the adsorbed substance, Xe is the amount of residual dissolved substance (residual amount at equilibrium), *a* and *b* are Langmuir constants, and *K* and *N* are Freundlich constants [58].

When equilibrium is reached, the absorbent no longer acts upon the oil; it just discolored. The bleaching process is performed at a contact range of 353–393°K (80–120°C) under vacuum for 20–40 min [62]. Usually, the treatment is done under a slight vacuum to prevent oxidation enhanced by the oil dispersion on earth particles.

The amount of adsorbent used ranges between 0.1 and 3 g/100 g depending on the crude oil quality. However, other higher-percentage bleaching materials can be used to meet final color requirements [62].

The pretreated oil is heated to 363–383K (90–110°C) under vacuum and afterward is mixed vigorously in the bleacher with the adsorbent (bleaching earth or/and activated carbon). After a retention time of 20–40 min, the oil-adsorbent mixture is filtered. Only filters in the separation of the oil-adsorbent were used. Because the centrifuges process is not suitable for this separation. Therefore, short filtration times, efficient filtration, and minimization of oil retention on the adsorbent matter are necessary. After this filtration, the oil is now ready for the deodorizing process.

#### 1.1.5. Dewaxing or Winterization

Waxes are esters of long-chain primary alcohols and long fatty acids. These acids have low solubility in oils, are high melting, and usually crystalline during the winter season at low and room temperature [29]. The wax generally does not negatively affect the functionality of the vegetable oil. The presence of wax affects the quality aspect of the oil, which gives it a cloudy appearance especially during the winter season. Its hazy appearance is due to the precipitation of dissolved waxes.

The dewaxing process is also called winterization. The term “winterization” appears as during winter when the temperature is low, waxes present in the oil crystallize, thereby giving a hazy appearance to the oil.

This process concerns a few types of oils rich with waxes, such as rice bran, canola, corn germ oil, sunflower [29], and olive pomace [70].

The dewaxing process includes three main processing steps. In the first one, the bleached oil should be heated to 328°K (55°C) to make sure the oil is completely liquid. Secondly, the oil is cooled slowly to 283–288 K (10–15°C). Ideally, the chilled oil is held for several hours at this temperature. In the third step, after finishing the crystallization, the cooled oil is pumped into a filter machine to separate the wax from vegetable oil. The filtration yields a clear liquid oil and the by-product waxes.

#### 1.1.6. Deodorization

The last stage in refining involves high temperature and requires a great care. A deodorized oil is devoid of any taste, even pleasant ones. Deodorization is a simple distillation process [31]. This operation allows for the elimination of free fatty acids and removes odors, different off-flavor components, contaminants (pesticides, light polycyclic aromatic hydrocarbons, and other volatile components) [30, 32]. Deodorization also removes residues of mineral oil saturated hydrocarbons (MOSH) and mineral oil aromatic hydrocarbons (MOAH) [71].

On the other hand, deodorization also has other negative effects. Among them, the important bioactive molecules such as tocopherols, squalene, sterols, and polyphenols may be removed in this step [30]. The most important are the destruction of some essential nutrients; unwanted side reactions like cis-trans-isomerization (the double bond isomerizes from cis to trans) [5, 21], conjugation, and polymerization [72]. Also, it sometimes generates other unwanted compounds such as 3-MCPD-esters [19, 72]. This last has been identified as a new class of oil-refining contaminants [73].

Careful execution of this process also improves the stability and color of the oil, whilst preserving its nutritional value. Deodorization is a vacuum steam distillation [31]. It involves no technological auxiliary and proceeds by a simple injection of water vapor into the oil. The process involves the passage of steam through layers of oil held in trays and heating to high temperatures 453–513 K (180–240°C) using a high-pressure steam boiler. Utilizing a very high vacuum, between 2 and 8 mmHg, the process removes undesirable odors caused by aldehydes, ketones, alcohols, short-chain fatty acids, and thermolabile pigments [31]. It is a steam stripping of taste and odor conveying substances that are more volatile than oil. The thermodynamic equilibrium of the oil and dissolved matter (taste releasing substance) is given by Raoult's law:

(6) $$\begin{array}{c} \frac{P_{vo}}{P_{v}}=\frac{V}{H}, \end{array}$$

where *P*_*vo*_ is the partial pressure of the volatile component dissolved at a given temperature, *P*_*v*_ is the partial pressure this component would have at this same temperature, *V* is the number of moles of the volatile components, and *H* is the number of moles of oil.

The obtained oil is subsequently conditioned under nitrogen to protect it from oxidation [74]. The careful execution of these processing steps ensures that fully refined oils possess good organoleptic and physicochemical qualities.

### 1.2. Physical Refining

**Figure 2:**
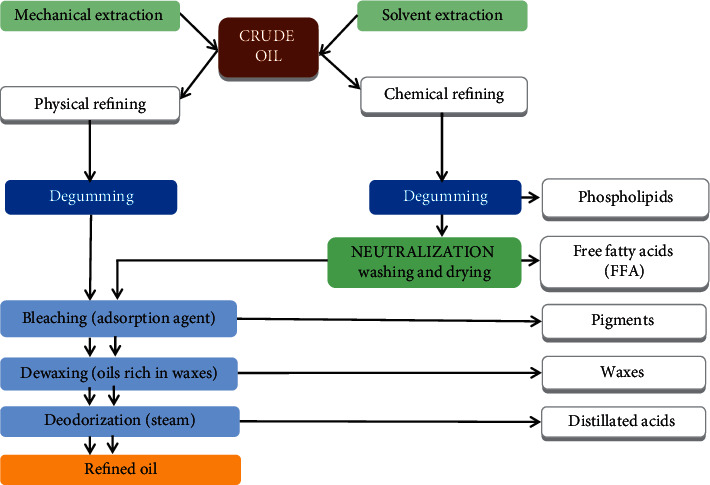
General overview of the chemical and physical refining process of crude oil.

The process consists of the same steps described in chemical refining, except for the alkali neutralization process [30]. The difference between chemical and physical refining is illustrated in Figure 2. Chemical refining consists of removing free fatty acids by adding caustic soda and separating the soap by centrifugation (mechanical separation) [29], while physical refining, in the last step, removes free fatty acids and other compounds by steam distillation. This process is also known as steam refining [29–32].

Physical refining of crude oils, therefore, overcomes the disadvantages of neutralization by sodium hydroxide [75]. Indeed, this process, which is deemed to be eco-friendly, minimizes liquid effluents generation [29, 30]. Another advantage of this process over chemical refining is that it is more economical (e.g., fewer chemicals used, lower investment cost, lesser energy input, and improved yield) [30, 32].

However, this process is not suitable for all types of oils since it is hypersensitive to the crude oil quality [30, 32]. Indeed, physical refining is used for oils with high acidity [75]. Considering the phospholipids content, Dumont and Narine [32] proposed two physical refining processes depending on the phospholipid content in the crude oil.

In general, physical refining includes the following three main processing steps:

1. Degumming to remove phosphatides
2. Bleaching and filtration to eliminate color pigments
3. Deodorization allows the elimination of free fatty acids and other volatile compounds

Sometimes the dewaxing process is added for types of oils rich in waxes (e.g., fatty acids with low solubility in oils and high-melting esters of fatty alcohols), such as corn, rice bran, canola, and sunflower oils [29]. The principle of physical refining includes the following three important steps. The first step consists of subjecting the oil to phosphoric acid in the short-mix chemical refining process. For degumming, it is the most important stage, and it must be performed carefully [31, 43].

To evaluate degumming efficiency for a given refined oil sample, an analysis test called “Degumming Efficiency” was developed. This test is determined via the following formula:

(7) $$\begin{array}{c} “\text{Degumming Efficiency}”\left(\frac{\text{g}}{100\text{ g}}\right)=\frac{\left(P_{0}-P_{d}\right)}{P_{0}}*100, \end{array}$$

where *P*_0_ is phospholipids (ppm) crude oil and *P*_*d*_ is phospholipids (ppm) degummed oil.

Bleaching (or decolorizing) is the second step. The objective is the same as that of chemical refining. The main aim of bleaching is to reduce the levels of some colored pigments (carotenoids and chlorophylls), but it also further removes residues of phosphatides traces, phospholipids traces contaminants, lipid peroxidation products, and other impurities [26, 74, 76]. The oil is then mixed with acid-activated bleaching earth or another adsorbent. The standard bleaching process temperature is 368–378°K (95–108°C). Spent adsorbent along with some precipitated carotenoids and other impurities are then removed by filtration. After this step, the oil is ready for deodorizing. The final step in the physical refining of oils is deacidification and deodorization. Oils are deodorized under the conditions described above for chemical refining.

The deodorization process has three main objectives:

1. Removal of volatile components such as free fatty acids, different off-flavors, contaminants (pesticides, light polycyclic aromatic hydrocarbons, etc.). However, it can eliminate partially some bioactive components (tocopherols, sterols, etc.)
2. Thermal bleaching of colored pigments and peroxides
3. To obtain better quality, a more acceptable aspect, and more flavor-stability during its shelf life

The deodorization process is fully determined by four process parameters: the amount of stripping steam, time, pressure, and temperature. Deodorization is usually carried out at high temperatures (>473°K) (>200°C) with low vacuum pressure. The use of high temperatures and vacuum often results in the formation of negative side products. The effects of process conditions on standard quality parameters and the nutritional quality of the oil are well documented in the literature [5, 19, 71, 72]. In conclusion, physical refining offers great several advantages over chemical refining for vegetable oils (Table 2).

## 2. Conclusions

A good understanding of edible oils chemistry along with their processing is very important to define quality product both for industry and for consumers. The objective of both types of refining is to get a better quality, a more acceptable aspect (limpidity), a lighter odor or color, a longer stability, and a good safety with the elimination of pollutants and FFA oxidation products. The problem is that most of these processes also remove the substances that contribute to the healthy properties of oils. This directly influences the stability of refined edible oils. Efficient refining processes should be developed for crude oils to remove undesirable compounds while doing the least possible damage to the interesting components at the same time and not generating higher levels of unwanted compounds.

## Figures and Tables

**Table 1:** Undesirable constituents in oil removed during refining.

Component	Origin	Effect
Free fatty acids	Hydrolysis of triglycerides	(i) Taste, smoke if heating
		(ii) Hydrolysis

Phosphatides (phospholipids)	Natural compounds	(i) Cloudy aspect
		(ii) Deposit a residue in the oil flavors
		(iii) Dark color if heating

Oxidation products	Oxidation of unsaturated fatty acids	(i) Undesirable flavors
		(ii) Stability
		(iii) Color—nutrition

Flavors	Natural compounds of seeds, autooxidation	(i) Odorous components
		(ii) Flavors

Waxes and pigments	Natural components of seeds	(i) Odorous components
		(ii) Flavors

Metals (iron and copper)	Technological pollution Pollution during storage transport and processing	(i) Oxidation catalysts
		(ii) Stability
Chemical pollutants		(i) Safety toxicity
Heavy metals		
Pesticides		
PAHs (B[a]P)		
Mycotoxins		
Dioxins		

**Table 2:** Advantages and disadvantage of chemical and physical refining.

	Physical refining	Chemical refining
Advantages	(i) Lower investment cost (ii) Fewer by-products generated (iii) Less energy consumed (iv) Fewer chemicals used (v) Environmentally friendly (vi) Improved yield oil	(i) Simple operating process (ii) Efficient process to eliminate FFA

Disadvantages	(i) Not suitable for all types of oils (ii) Requires high temperature and vacuum (iii) Can form unwanted products	(i) Production of side products (ii) Expensive process (iii) Removes a high percentage of oil (iv) Can form unwanted products

## Data Availability

The data analyzed during this review were included in the document; therefore, no additional data are available.

## References

[1] Brahmi F., Haddad S., Bouamara K. (2020). Comparison of chemical composition and biological activities of Algerian seed oils of *Pistacia lentiscus* L., *Opuntia ficus indica* (L.) mill. and *Argania spinosa* L. skeels. *Industrial Crops and Products*.

[2] Vidrih R., Vidakovič S., Abramovič H. (2010). Biochemical parameters and oxidative resistance to thermal treatment of refined and unrefined vegetable edible oils. *Czech Journal of Food Sciences*.

[3] Ying Q., Rudzińska M., Grygier A., Przybylski R. (2020). Determination of triacylglycerols by HTGC-FID as a sensitive tool for the identification of rapeseed and olive oil adulteration. *Molecules*.

[4] Gnanaprakasam A., Sivakumar V. M., Surendhar A., Thirumarimurugan M., Kannadasan T. (2013). Recent strategy of biodiesel production from waste cooking oil and process influencing parameters: a review. *Journal of Energy*.

[5] Gharby S., Guillaume D., Elibrahimi M., Charrouf Z. (2021). Physico-chemical properties and sensory analysis of deodorized argan oil. *ACS Food Science & Technology*.

[6] Chew S.-C., Tan C.-P., Long K., Nyam K.-L. (2016). Effect of chemical refining on the quality of kenaf (*Hibiscus cannabinus*) seed oil. *Industrial Crops and Products*.

[7] Gharby S., Harhar H., Mamouni R., Matthäus B., Ait Addi E. H., Charrouf Z. (2016). Chemical characterization and kinetic parameter determination under rancimat test conditions of four monovarietal virgin olive oils grown in Morocco. *Ocl*.

[8] Aliyar-Zanjani N., Piravi-Vanak Z., Ghavami M. (2019). Study on the effect of activated carbon with bleaching earth on the reduction of polycyclic aromatic hydrocarbons (PAHs) in bleached soybean oil. *Grasas y Aceites*.

[9] Lamas D. L., Constenla D. T., Raab D. (2016). Effect of degumming process on physicochemical properties of sunflower oil. *Biocatalysis and Agricultural Biotechnology*.

[10] Chew S. C., Nyam K. L. (2020). Refining of edible oils. *Lipids and Edible Oils*.

[11] Evrard J., Pagès-Xatart-Pares X., Argenson C., Morin O. (2007). Procédés d’obtention et compositions nutritionnelles des huiles de tournesol, olive et colza. *Cahiers de Nutrition et de Dietetique*.

[12] Sánchez‐Arévalo C. M., Olmo‐García L., Fernández‐Sánchez J. F., Carrasco‐Pancorbo A. (2020). Polycyclic aromatic hydrocarbons in edible oils: an overview on sample preparation, determination strategies, and relative abundance of prevalent compounds. *Comprehensive Reviews in Food Science and Food Safety*.

[13] Zio S., Cisse H., Zongo O. (2020). The oils refining process and contaminants in edible oils: a review. *Journal of Food Technology Research*.

[14] Chew S.-C., Tan C.-P., Nyam K.-L. (2017). Application of response surface methodology for optimizing the deodorization parameters in chemical refining of kenaf seed oil. *Separation and Purification Technology*.

[15] Lacoste F. (2014). Undesirable substances in vegetable oils: anything to declare?. *Ocl*.

[16] Bonwick G. A., Birch C. S. (2019). European regulation of process contaminants in food. *Mitigating Contamination from Food Processing*.

[17] Programme Mixte FAO/OMS Sur Les Normes Alimentaires (2019). *Commission Du Codex Alimentarius. Quarante-Deuxième Session*.

[18] Lucci P., Bertoz V., Pacetti D., Moret S., Conte L. (2020). Effect of the refining process on total hydroxytyrosol, tyrosol, and tocopherol contents of olive oil. *Foods*.

[19] Arris F. A., Thai V. T. S., Manan W. N., Sajab M. S. (2020). A revisit to the formation and mitigation of 3-chloropropane-1,2-diol in palm oil production. *Foods*.

[20] Harhar H., Gharby S., Kartah B., Pioch D., Guillaume D., Charrouf Z. (2014). Effect of harvest date of Argania spinosa fruits on Argan oil quality. *Industrial Crops and Products*.

[21] Fang B., Zhang M., Shen Y. M. (2017). Importance of the higher retention of tocopherols and sterols for the oxidative stability of soybean and rapeseed oils. *Journal of Food Science & Technology*.

[22] Verhé R., Verleyen T., Van Hoed V., De Greyt W. (2006). Influence of refining of vegetable oils on minor components. *Journal of Oil Palm Research*.

[23] Ghazani S. M., Marangoni A. G. G. (2013). Minor components in canola oil and effects of refining on these constituents: a review. *Journal of the American Oil Chemists Society*.

[24] Gotor A. A., Rhazi L. (2016). Effects of refining process on sunflower oil minor components: a review. *OCL*.

[25] Chew S. C., Ali M. A. (2021). Recent advances in ultrasound technology applications of vegetable oil refining. *Trends in Food Science & Technology*.

[26] Vaisali C., Charanyaa S., Belur P. D., Regupathi I. (2014). Refining of edible oils: a critical appraisal of current and potential technologies. *International Journal of Food Science and Technology*.

[27] Ortega-García J., Gámez-Meza N., Noriega-Rodriguez J. A. (2006). Refining of high oleic safflower oil: effect on the sterols and tocopherols content. *European Food Research and Technology*.

[28] Gharby S., Hajib A., Ibourki M. (2021). Induced changes in olive oil subjected to various chemical refining steps: a comparative study of quality indices, fatty acids, bioactive minor components, and oxidation stability kinetic parameters. *Chemical Data Collections*.

[29] Manjula S., Subramanian R. (2006). Membrane technology in degumming, dewaxing, deacidifying, and decolorizing edible oils. *Critical Reviews in Food Science and Nutrition*.

[30] Tasan M., Demirci M. (2005). Total and individual tocopherol contents of sunflower oil at different steps of refining. *European Food Research and Technology*.

[31] Hussain Sherazi S. T., Mahesar S. A., Sirajuddin (2016). Vegetable oil deodorizer distillate: a rich source of the natural bioactive components. *Journal of Oleo Science*.

[32] Dumont M.-J., Narine S. S. (2007). Soapstock and deodorizer distillates from North American vegetable oils: review on their characterization, extraction and utilization. *Food Research International*.

[33] Delgado A., Al-Hamimi S., Ramadan M. F. (2020). Contribution of tocols to food sensorial properties, stability, and overall quality. *Journal of Food Quality*.

[34] Giriprasad R H. S., Goswami M. (2013). Animal fat-processing and its quality control. *Journal of Food Processing & Technology*.

[35] Van Nieuwenhuyzen W., Tomás M. C. (2008). Update on vegetable lecithin and phospholipid technologies. *European Journal of Lipid Science and Technology*.

[36] Dijkstra A. J. (2017). About water degumming and the hydration of non‐hydratable phosphatides. *European Journal of Lipid Science and Technology*.

[37] Wibisono Y., Nugroho W. A., Chung T.-W. (2014). Dry degumming of corn-oil for biodiesel using a tubular ceramic membrane. *Procedia Chemistry*.

[38] Zufarov O., Schmidt S., Sekretár S. (2008). Degumming of rapeseed and sunflower oils. *Acta Chimica Slovaca*.

[39] De B. K., Patel J. D. (2010). Effect of different degumming processes and some nontraditional neutralizing agent on refining of RBO. *Journal of Oleo Science*.

[40] Issaoui M., Delgado A. M., Ramadan M. F. (2019). Grading, labeling and standardization of edible oils. *Fruit Oils’: Chemistry and Functionality*.

[41] Clausen K. (2001). Enzymatic oil-degumming by a novel microbial phospholipase. *European Journal of Lipid Science and Technology*.

[42] Dijkstra A. J. (2010). Enzymatic degumming. *European Journal of Lipid Science and Technology*.

[43] Yang J.-G., Wang Y.-H., Yang B., Mainda G., Guol Y. (2006). Degumming of vegetable oil by a new microbial lipase. *Food Technology and Biotechnology*.

[44] Sadeghi M. (2010). Purification of soybean oil with phospholipase Al. *Theoretical and Experimental Chemistry*.

[45] Sampaio K. A., Zyaykina N., Wozniak B., Tsukamoto J., Greyt W. D., Stevens C. V. (2015). Enzymatic degumming: degumming efficiency versus yield increase. *European Journal of Lipid Science and Technology*.

[46] Yang B., Zhou R., Yang J.-G., Wang Y.-H., Wang W.-F. (2008). Insight into the enzymatic degumming process of soybean oil. *Journal of the American Oil Chemists Society*.

[47] Jiang X., Chang M., Wang X., Jin Q., Wang X. (2014). The effect of ultrasound on enzymatic degumming process of rapeseed oil by the use of phospholipase A1. *Ultrasonics Sonochemistry*.

[48] Gharby S., Harhar H., Farssi M., Ait Taleb A., Guillaume D., Laknifli A. (2018). Influence of roasting olive fruit on the chemical composition and polycyclic aromatic hydrocarbon content of olive oil. *Ocl*.

[49] Essid K., Chtourou M., Trabelsi M., Frikha M. H. (2009). Influence of the neutralization step on the oxidative and thermal stability of acid olive oil. *Journal of Oleo Science*.

[50] Gertz C., Parkash Kochhar S. (2001). A new method to determine oxidative stability of vegetable fats and oils at simulated frying temperature. *Oléagineux, Corps Gras, Lipides*.

[51] Ruiz-Méndez M. V., Márquez-Ruiz G., Dobarganes M. C. (1997). Relationships between quality of crude and refined edible oils based on quantitation of minor glyceridic compounds. *Food Chemistry*.

[52] Patel V. R., Dumancas G. G., Kasi Viswanath L. C., Maples R., Subong B. J. (2016). Castor oil: properties, uses, and optimization of processing parameters in commercial production. *Lipid Insights*.

[53] Piloto-Rodríguez V. S., Melo1 E. A., Goyos-Pérez L. (2014). Conversion of by-products from the vegetable oil industry into biodiesel and its use in internal combustion engines: a review. *Brazilian Journal of Chemical Engineering*.

[54] Pereda Marín J., BarrigaMateos F., Mateo y P. Á. . (2003). Aprovechamiento de las oleinasresidualesprocedentes del proceso de refinadode los aceitesvegetales comestibles, para la fabricación de biodiesel. *Grasas y Aceites*.

[55] Luxem F J M. B. K., Ching T. H., Jei-Fu S. (2008). Biocatalysis and bioenergy. *Biodiesel from Acidulated Soapstock (Acid Oil)*.

[56] Haslenda H., Jamaludin M. Z. (2011). Industry to Industry By-products Exchange Network towards zero waste in palm oil refining processes. *Resources, Conservation and Recycling*.

[57] Monte M. L., Monte M. L., Pohndorf R. S., Crexi V. T., Pinto L. A. A. (2015). Bleaching with blends of bleaching earth and activated carbon reduces color and oxidation products of carp oil. *European Journal of Lipid Science and Technology*.

[58] Liu Y., Huang J., Wang X. (2008). Adsorption isotherms for bleaching soybean oil with activated attapulgite. *Journal of the American Oil Chemists Society*.

[59] Zschau W. (2001). Bleaching of edible fats and oils. *European Journal of Lipid Science and Technology*.

[60] Sabah E., Çinar M., Çelik M. S. (2007). Decolorization of vegetable oils: adsorption mechanism of *β*-carotene on acid-activated sepiolite. *Food Chemistry*.

[61] Amari A., Gannouni H., Khan M., Almesfer M., Elkhaleefa A., Gannouni A. (2018). Effect of structure and chemical activation on the adsorption properties of green clay minerals for the removal of cationic dye. *Applied Sciences*.

[62] Usman M. A., Ekwueme V. I., Alaje T. O., Mohammed A. O. (2012). Characterization, acid activation, and bleaching performance of ibeshe clay, lagos, Nigeria. *ISRN Ceramics*.

[63] Javed S. H., Zahir A., Khan A., Afzal S., Mansha M. (2018). Adsorption of mordant red 73 dye on acid activated bentonite: kinetics and thermodynamic study. *Journal of Molecular Liquids*.

[64] Gong Z., Alef K., Wilke B.-M., Li P. (2007). Activated carbon adsorption of PAHs from vegetable oil used in soil remediation. *Journal of Hazardous Materials*.

[65] Ma Y., Shi L., Liu Y., Lu Q. (2017). Effects of neutralization, decoloration, and deodorization on polycyclic aromatic hydrocarbons during laboratory-scale oil refining process. *Journal of Chemistry*.

[66] Amzad Hossain M., Salehuddin S. M. (2012). Polycyclic aromatic hydrocarbons (PAHs) in edible oils by gas chromatography coupled with mass spectroscopy. *Arabian Journal of Chemistry*.

[67] Tongpoothorn W., Sriuttha M., Homchan P., Chanthai S., Ruangviriyachai C. (2011). Preparation of activated carbon derived from Jatropha curcas fruit shell by simple thermo-chemical activation and characterization of their physico-chemical properties. *Chemical Engineering Research and Design*.

[68] Vargas J. E., Gutierrez L. G., Moreno-Piraján J. C. (2010). Preparation of activated carbons from seeds of *Mucuna mutisiana* by physical activation with steam. *Journal of Analytical and Applied Pyrolysis*.

[69] Mohamed A. R., Mohammadi M., Darzi G. N. (2010). Preparation of carbon molecular sieve from lignocellulosic biomass: a review. *Renewable and Sustainable Energy Reviews*.

[70] Omar S., Girgis B., Taha F. (2003). Carbonaceous materials from seed hulls for bleaching of vegetable oils. *Food Research International*.

[71] Siragakis G., Antonopoulos K., Valet N., Spiratos D. (2006). Olive oil and pomace olive oil processing. *Grasas y Aceites*.

[72] Stauff A., Schnapka J., Heckel F., Matissek R. (2020). Mineral oil hydrocarbons (MOSH/MOAH) in edible oils and possible minimization by deodorization through the example of cocoa butter. *European Journal of Lipid Science and Technology*.

[73] Hafidi A., Pioch D., Ajana H. (2005). Membrane-based simultaneous degumming and deacidification of vegetable oils. *Innovative Food Science & Emerging Technologies*.

[74] Cheng Z., Liu G., Wang L. (2017). Glycidyl fatty acid esters in refined edible oils: a review on formation, occurrence, analysis, and elimination methods. *Comprehensive Reviews in Food Science and Food Safety*.

[75] Di Giovacchino L., Mucciarella M. R., Costantini N., Ferrante M. L., Surricchio G. (2002). Use of nitrogen to improve stability of virgin olive oil during storage. *Journal of the American Oil Chemists Society*.

[76] Silva S. M., Sampaio K. A., Ceriani R. (2014). Effect of type of bleaching earth on the final color of refined palm oil. *Lebensmittel-Wissenschaft und -Technologie- Food Science and Technology*.

